# Influenza Vaccination Willingness, Uptake, and Behavioral Drivers Among Adults Aged ≥60 Years in Henan Province: A BeSD-Based Survey with Registry Follow-Up

**DOI:** 10.3390/vaccines14070605

**Published:** 2026-07-09

**Authors:** Jun Li, Xinyang Li, Kaichao Yang, Yuxia Yun, Yanyan Yang, Lijun Deng, Zunshui Li, Xiaoyang Wang, Xiaoxiao Zhang, Lubin Shi, Binghui Du, Yanfang Ji, Yonghao Guo, Yanyang Zhang, Shuaiyin Chen

**Affiliations:** 1 Immunization Prevention and Planning Department, Henan Center for Disease Control and Prevention, Zhengzhou 450016, China; henan_epi2@163.com (J.L.); yangkc186@163.com (K.Y.); wxy6424@163.com (X.W.); xiao5541@126.com (X.Z.); shilubin3386@163.com (L.S.); dbh1101@163.com (B.D.); jiyanfangyx@163.com (Y.J.); cdcgyh@163.com (Y.G.); 2 Henan Preventive Medicine Institute, Zhengzhou 450016, China; a1322468989@163.com; 3 School of Public Health, Zhengzhou University, Zhengzhou 450001, China; sychen@zzu.edu.cn; 4 Pharmacy and Medical Equipment Department, Henan Center for Disease Control and Prevention, Zhengzhou 450016, China; 5 Immunization Planning Section, Puyang Center for Disease Control and Prevention, Puyang 457000, China; yunyuxia518@163.com; 6 Xinyang Center for Disease Control and Prevention, Xinyang 464000, China; jerry12799@163.com; 7 Immunization Planning Department, Zhengzhou Center for Disease Control and Prevention, Zhengzhou 450007, China; denglijun1188@163.com; 8 Immunization Planning Department, Sanmenxia Center for Disease Control and Prevention, Sanmenxia 472000, China; smxjmk@163.com

**Keywords:** Behavioral and Social Drivers (BeSD), adults aged ≥60 years, influenza vaccine, vaccination willingness, vaccination uptake, intention-behavior gap, instant educational intervention

## Abstract

Objectives: To identify factors influencing influenza vaccination willingness and uptake among adults aged ≥60 years in Henan Province and to evaluate the effect of a brief educational intervention on vaccination willingness and behavior. Methods: In September 2024, a cross-sectional survey based on the Behavioral and Social Drivers (BeSD) framework was conducted among adults aged ≥60 years across five counties in Henan. For participants without baseline willingness, a 3 min one-on-one educational intervention was delivered. In May 2025, following the end of the 2024–2025 influenza vaccination season (which runs from 1 October to 31 March in Henan Province), we retrieved vaccination records for all participants from the Henan Provincial Immunization Information System. This system captures all influenza vaccinations administered at designated vaccination clinics across the province. To ensure completeness for doses administered outside the provincial system (e.g., in other provinces or at private healthcare facilities), we conducted telephone follow-up interviews with all participants whose baseline vaccination intention was inconsistent with their actual vaccination behavior (i.e., willing but unvaccinated or unwilling but vaccinated). During these interviews, for those who reported receiving the vaccine outside Henan Province or at private facilities, we inquired about the specific date and location of vaccination to supplement the registry data. We also explored the reasons behind the intention–behavior discrepancy. For these participants, we requested vaccination certificates or other supporting documentation to confirm their vaccination status. Results: Baseline vaccination willingness was 68.20% (1630/2390), whereas the actual vaccination rate was only 6.95% (166/2390), yielding a willingness-to-behavior conversion rate of 9.51% (155/1630) among those with baseline willingness. Of the 760 participants without baseline willingness, 543 (71.45%) completed the 3 min one-on-one instant educational intervention and the follow-up assessment; the remaining 217 were excluded due to refusal or loss to follow-up. Among these 543 completers, 46 (8.47%) became willing to vaccinate, and eight (1.47%) were subsequently vaccinated. Multivariate analysis identified the social processes dimension as the strongest correlate of both willingness (OR = 1.38 per 1-point increase, 95% CI: 1.33–1.44) and uptake (OR = 1.12, 95% CI: 1.03–1.22). Urban residence was associated with higher willingness (OR = 1.41, 95% CI: 1.12–1.78) and higher uptake (OR = 1.64, 95% CI: 1.11–2.42). Current smokers had a significantly lower uptake than never smokers (OR = 0.43, 95% CI: 0.22–0.85). Among the 11 participants without baseline willingness who were eventually vaccinated (eight from the intervention group and three from the non-intervention group), family/friend influence (63.64%, 7/11) and physician recommendation (36.36%, 4/11) were the primary drivers. For those with willingness but no action (*n* = 1475), the main barriers were perceived good health (33.29%), high vaccine cost (27.12%), and lack of time (26.31%). Conclusions: Influenza vaccination among older adults in Henan exhibits a “high willingness, low conversion” pattern, with social processes as the strongest driver bridging the intention–behavior gap. A brief educational intervention improved willingness but failed to translate into meaningful uptake, underscoring that knowledge transfer alone is insufficient. We recommend a multi-component strategy that (1) mobilizes family members and community doctors as trusted vaccine advocates; (2) leverages family and village doctor networks to reduce urban–rural disparities; (3) counters the “perceived good health” barrier with age-specific risk communication; and (4) integrates vaccine recommendations into routine care for high-risk groups, particularly frequent outpatient attendees and smokers.

## 1. Introduction

Influenza (flu) is an acute respiratory infectious disease caused by influenza viruses, with elderly individuals facing elevated risks of severe illness and mortality [1]. Individuals aged ≥60 years bear a heavier burden of influenza-related outpatient/inpatient visits and deaths compared to other age groups [2]. From 2005 to 2019, China’s influenza-associated all-cause mortality rate was 14.33 per 100,000 population, rising to 122.79 per 100,000 population among those aged ≥65 years [3]. Influenza vaccination is the most economical and effective preventive measure. A meta-analysis reported that vaccination confers 58% (95% CI: 48–66%) protection against influenza-like illness and 40% (95% CI: 30–50%) protection against confirmed influenza in the elderly [4]. While studies indicate high vaccination willingness among adults ≥60 years [5], China’s elderly influenza vaccination rate remains below 10% [6].

According to a recent analysis of Henan’s Immunization Information System, the annual average vaccination rates among adults aged ≥50 years from 2020 to 2023 were 3.00% for the influenza vaccine, 0.30% for the pneumococcal vaccine, and only 0.06% for the herpes zoster vaccine [7]. While influenza vaccination showed an increasing trend—rising from 2.62% to 3.63% over the study period—these rates remain substantially lower than those in Beijing (14.14–18.83%) and Zhejiang Province (20.41–21.39%) [8]. Henan has no province-wide free influenza vaccination policy for older adults, yet Xinxiang City has been an exception: since 2008, it has provided free vaccination to local residents aged ≥65 years, achieving a markedly higher rate of 12.04% among adults aged ≥60 during 2020–2022 versus the provincial average of 2.77% [9].

To address low vaccination rates, the World Health Organization (WHO) released the Behavioral and Social Drivers (BeSD) framework in 2022, which explains vaccination behavior through four dimensions: thinking and feeling (e.g., risk perception, vaccine confidence), social processes (e.g., recommendations from family, friends, and healthcare providers), practical issues (e.g., cost, accessibility, and convenience), and motivation (i.e., vaccination willingness) [10]. In 2023, the Chinese Center for Disease Control and Prevention (China CDC) developed an elderly influenza vaccination willingness questionnaire based on the BeSD framework and validated it in Jiaozuo, Henan, with good reliability [11].

However, existing studies lack systematic exploration of the willingness-to-behavior conversion mechanism and the impact of interventions on translating willingness into action. Most previous research has focused either on identifying correlates of vaccination intention or on evaluating policy effects, with limited attention to the dynamic process through which intentions are translated—or fail to be translated—into actual behavior. This study is the first to investigate influenza vaccination willingness among older adults aged ≥60 years in Henan using the BeSD framework, incorporating instant health education interventions for individuals without baseline willingness and following up on their vaccination behavior. By adopting an “instant intervention + follow-up evaluation” design, this study assesses how BeSD dimensions moderate the willingness-to-behavior conversion, providing evidence for targeted intervention strategies.

## 2. Subjects and Methods

### 2.1. Study Design

This study employed a hybrid design consisting of (1) a cross-sectional survey to assess baseline vaccination willingness and its associated factors based on the BeSD framework; (2) a non-randomized, 3 min one-on-one instant educational intervention among participants with no baseline willingness; and (3) a post hoc follow-up combining immunization registry data with baseline willingness data and follow-up interview data to assess actual vaccination uptake and the factors influencing willingness-to-behavior conversion among participants whose willingness was inconsistent with their actual behavior.

### 2.2. Study Population

Stratified by per capita GDP (high, medium, and low) based on the most recent statistical yearbook data at the time of study design, five counties/districts were randomly selected from the 157 county-level administrative units in Henan Province: Xinzheng City (Zhengzhou), Puyang County (Puyang), Pingqiao District (Xinyang), Mianchi County (Sanmenxia), and Yongcheng City (Shangqiu). These counties/districts are geographically distributed across northern, central, and southern Henan to ensure regional representativeness.

From each selected county/district, one urban area and one rural area were randomly selected using a random number table from all subdistrict/township units. Across the five counties/districts, this sampling frame comprised 17 subdistricts and 77 townships/towns, totaling 94 subdistrict/township units (13 in Xinzheng, 22 in Puyang County, 16 in Pingqiao District, 12 in Mianchi County, and 31 in Yongcheng City). Subsequently, one community from the selected urban area and one administrative village from the selected rural area were randomly selected from all communities/villages within each chosen subdistrict/township. Community and village doctors assisted the field investigators in recruiting participants through convenience sampling for one-on-one, face-to-face interviews.

Inclusion criteria: (1) local residents with ≥6 months of continuous residence; (2) adults aged ≥60 years; (3) informed consent and voluntarily participated.

Exclusion criteria: (1) cognitive impairment precluding normal communication; (2) poor compliance preventing follow-up. Poor compliance was defined as (a) inability to complete the baseline questionnaire or (b) refusal to participate in the follow-up interview.

### 2.3. Study Methods

#### 2.3.1. Sample Size Calculation

The sample size for this study was calculated based on the formula for cross-sectional surveys. Based on previous literature and local circumstances, the expected influenza vaccination willingness rate among people aged ≥60 years was set at 70%, with a margin of error of 3% and a 95% confidence level. A design effect of 1.5 was applied to account for complex sampling, and a 20% follow-up loss rate was considered. Under these parameters, the calculated minimum required sample size was 1680 participants. In practice, a total of 2390 participants were finally enrolled to ensure sufficient statistical power for subgroup analyses, which met the sample size requirement.

#### 2.3.2. Survey Content and Procedures

Baseline survey was conducted in September 2024 by uniformly trained investigators through one-on-one interviews. Content included: (1) Demographics (age, gender, education, income, living condition, etc.). (2) Health status and behaviors (self-rated health, smoking history, annual medical visits, chronic disease history, etc.). (3) BeSD dimensions (thinking and feeling: 7 items, total score 28; social processes: 5 items, total score 20; practical issues: 4 items, total score 16; and 4-point Likert scale). (4) All participants took the baseline vaccination willingness survey. (5) For participants with no baseline vaccination willingness, a 3 min one-on-one instant intervention was carried out. The intervention was offered to all participants who expressed no baseline willingness and provided additional informed consent; this was a non-randomized component, as randomization was not feasible in the community setting. Investigators delivered face-to-face health education using materials developed based on the China Influenza Vaccine Prevention and Immunization Technical Guidelines (2023–2024), specifically explaining the influenza morbidity risk, severity of complications, and disease burden among people aged ≥60 years, as well as vaccine safety and the importance of vaccination. (6) In May 2025, following the end of the 2024–2025 influenza vaccination season (which runs from 1 October to 31 March in Henan Province), we retrieved vaccination records for all participants from the Henan Provincial Immunization Information System. This system captures all influenza vaccinations administered at designated vaccination clinics across the province. We conducted telephone follow-up interviews with all participants whose baseline vaccination intention was inconsistent with their actual vaccination behavior (i.e., willing but unvaccinated or unwilling but vaccinated). During these interviews, for those who reported receiving the vaccine outside Henan Province or at private facilities, we inquired about the specific date and location of vaccination to supplement the registry data. We also explored the reasons behind the intention–behavior discrepancy.

#### 2.3.3. Quality Control

A pilot survey was conducted in September 2024 among 120 older adults in Mianchi County, Sanmenxia (not included in the final sample), to assess the clarity of the questionnaire and the feasibility of the 3 min intervention script. Based on pilot feedback, we revised several items. Investigators received one day of uniform training. Data were double-entered and cross-checked for accuracy.

### 2.4. Statistical Analysis

Analyses were performed using the R language. Continuous data are presented as “mean ± standard deviation”, with group comparisons via *t*-tests. Categorical data are presented as “n (%)”, with group comparisons via χ^2^ tests. Multivariate logistic regression (stepwise selection: α-in = 0.05, α-out = 0.10) identified independent factors of vaccination willingness and behavior; model fit was assessed via the Hosmer–Lemeshow test. Paired *t*-tests compared knowledge and attitude scores before and after intervention, α = 0.05. The Shapiro–Wilk test was used to assess normality; continuous variables with normal distribution were compared using *t*-tests; otherwise, Mann–Whitney U tests were used. Internal consistency was assessed using Cronbach’s α, and construct validity via confirmatory factor analysis (CFA) with RMSEA, SRMR, CFI, and TLI.

## 3. Results

### 3.1. Basic Characteristics of Study Subjects

**Figure 1:**
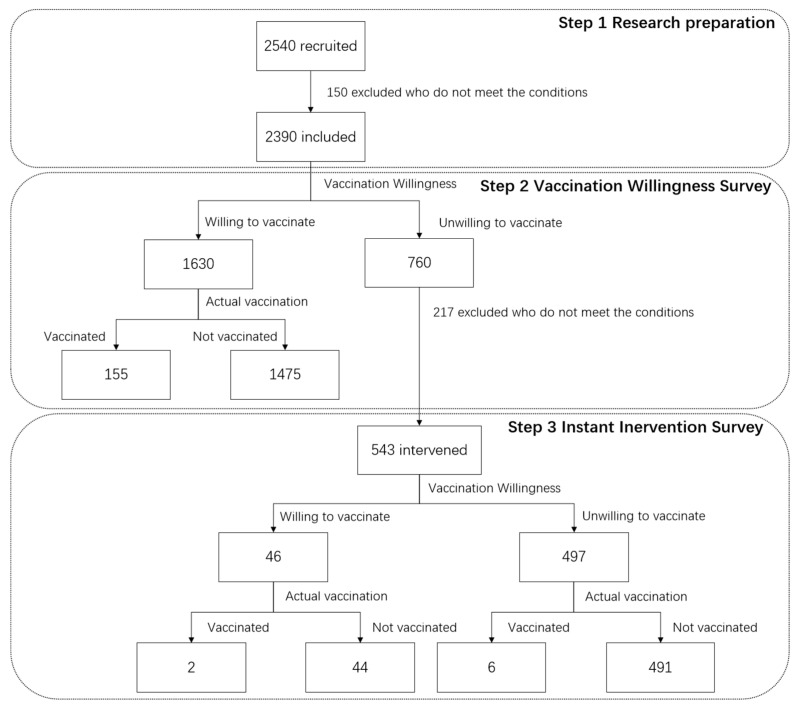
Flowchart.

A total of 2540 individuals were recruited; 150 were excluded (35 for age mismatch, 115 for follow-up loss, incorrect ID, or missing data), leaving 2390 participants, see Figure 1. Rural residents accounted for 50.88% (1216/2390), males 43.10% (1030/2390), and mean age was 70.42 ± 6.73 years old. Most lived with partners/children (86.78%, 2074/2390), had junior high school or lower education (85.44%, 2042/2390), and had no/low income (monthly income < 1000 yuan: 77.53%, 1853/2390). Urban and rural participants differed significantly in education and monthly income (*p* < 0.001), but other characteristics were balanced. Specific data could be seen in Table 1 and Table 2.

### 3.2. Questionnaire Reliability and Validity

The scale’s Cronbach α coefficient was 0.91; subscales for thinking and feeling (0.88), social processes (0.88), and practical issues (0.73) demonstrated good reliability. Confirmatory factor analysis showed acceptable model fit (RMSEA = 0.079, SRMR = 0.048, CFI = 0.928, and TLI = 0.915), with all item loadings statistically significant (*p* < 0.001), supporting the construct validity of the BeSD questionnaire.

### 3.3. Vaccination Willingness and Uptake

During the 2024–2025 influenza season (1 October 2024 to 31 March 2025), baseline vaccination willingness was 68.20% (1630/2390, 95% CI: 66.29–70.07%). Based on immunization registry records supplemented through follow-up telephone interviews, the final actual vaccination rate was 6.95% (166/2390, 95% CI: 5.96–8.04%). Among those with willingness, 9.51% (155/1630, 95% CI: 8.12–11.04%) were vaccinated; among those without willingness, 1.45% (11/760, 95% CI: 0.73–2.58%) were vaccinated (χ^2^ = 52.12, *p* < 0.01).

Among the 760 participants without baseline willingness, 543 (71.45%) completed the 3 min one-on-one instant educational intervention and the follow-up assessment; the remaining 217 were excluded (98 refused the intervention, 119 were lost to follow-up). Among these 543 completers, 46 (8.47%, 95% CI: 6.30–11.12%) became willing to vaccinate after the intervention. Vaccination willingness before (0/543) and after (46/543) the intervention differed significantly (McNemar’s test, *p* < 0.01). Ultimately, eight of the 543 completers (1.47%, 95% CI: 0.64–2.88%) were vaccinated.

Follow-up of willingness–behavior mismatch: Among the 1475 participants with baseline willingness who were not vaccinated, the main barriers were perceived good health (33.29%, 491/1475), high vaccine cost (27.12%, 400/1475), lack of time (26.31%, 388/1475), and medical contraindications (11.53%, 170/1475). Among the 11 initially unwilling participants who were eventually vaccinated, the primary drivers were family/friend influence (63.64%, 7/11), clinical physician recommendation (36.36%, 4/11), improved vaccine knowledge (27.27%, 3/11), and vaccination clinic staff recommendation (18.18%, 2/11). These findings, based on a small subsample (*n* = 11), should be interpreted cautiously.

### 3.4. Factors Associated with Vaccination Willingness

Univariate analysis showed that vaccination willingness varied by age group, with the highest rate in the 65–69 age group (71.66%, 445/621, and 95% CI: 68.04–75.08%), followed by the 60–64 age group (70.06%, 358/511, and 95% CI: 65.99–73.89%); the difference was statistically significant (χ^2^ = 8.86, *p* = 0.03). Willingness increased with education level (χ^2^ = 49.91, *p* < 0.01), ranging from 62.52% (522/835, 95% CI: 59.16–65.79%) among those with primary school or below to 86.49% (64/74, 95% CI: 76.85–93.00%) among those with college or above. Urban residents had higher willingness than rural residents (75.64% vs. 61.02%, χ^2^ = 58.86, and *p* < 0.01). Willingness increased with monthly income (χ^2^ = 58.27, *p* < 0.01), ranging from 62.69% (662/1056, 95% CI: 59.73–65.58%) among those with 0–1000 yuan to 84.08% (301/358, 95% CI: 79.87–87.76%) among those with ≥2000 yuan. Those living with partners/children had a higher willingness than those living alone (69.29% vs. 61.08%, χ^2^ = 11.15, and *p* < 0.01). No significant differences were observed by gender, county, annual medical visits, chronic disease status, smoking, or self-rated health.

Participants with willingness scored higher on thinking and feeling (22.13 ± 4.32 vs. 19.49 ± 4.41, t = 13.84, and *p* < 0.01), social processes (15.98 ± 3.22 vs. 11.45 ± 3.67, t = 29.21, and *p* < 0.01), and practical issues (12.41 ± 2.52 vs. 10.12 ± 2.46, t = 20.86, and *p* < 0.01) than those without.

Multivariate logistic regression (variable assignments shown in Supplementary Table S1) showed that urban residence was associated with higher willingness (OR = 1.41, 95% CI: 1.12–1.78, and *p* < 0.01); each one-point increase in the social processes score was associated with 38% higher odds of willingness (OR = 1.38, 95% CI: 1.33–1.44, and *p* < 0.01); each one-point increase in the practical issues score was associated with 12% higher odds of willingness (OR = 1.12, 95% CI: 1.06–1.19, and *p* < 0.01). The Hosmer–Lemeshow test was statistically significant (χ^2^ = 19.27, *p* = 0.01), suggesting suboptimal model calibration; results should be interpreted with caution. Specific data could be seen in Table 3.

### 3.5. Factors Associated with Willingness to Behavior Conversion

Among 1630 participants with willingness, 155 were vaccinated. Univariate analysis showed that urban residents had higher conversion rates than rural residents (11.37%, 101/888, 95% CI: 9.37–13.65% vs. 7.28%, 54/742, 95% CI: 5.54–9.38%, χ^2^ = 7.88, and *p* < 0.01). Conversion rates varied significantly by annual medical visit frequency (χ^2^ = 20.63, *p* < 0.01), with the highest rate among those with ≥11 visits (21.21%, 14/66, and 95% CI: 12.05–33.14%) and the lowest among those with 2–5 visits (5.92%, 33/557, and 95% CI: 4.12–8.24%). Smoking status was also associated with conversion (χ^2^ = 7.48, *p* = 0.02): never smokers (10.12%, 117/1156, and 95% CI: 8.44–12.02%) and former smokers (11.02%, 28/254, and 95% CI: 7.45–15.59%) had higher rates than current smokers (4.55%, 10/220, and 95% CI: 2.21–8.23%). No significant differences were observed by age, gender, education, income, county, living arrangement, chronic disease status, or self-rated health.

Multivariate logistic regression (variable assignments shown in Supplementary Table S2) showed that urban residence was associated with higher willingness-to-behavior conversion (OR = 1.64, 95% CI: 1.11–2.42, and *p* < 0.01); current smokers had lower conversion than never smokers (OR = 0.43, 95% CI: 0.22–0.85, and *p* = 0.02); participants with ≥11 annual medical visits had higher conversion than those with ≤1 visit (OR = 2.67, 95% CI: 1.33–5.33, and *p* < 0.01); participants with 2–5 annual visits had lower conversion than those with ≤1 visit (OR = 0.59, 95% CI: 0.39–0.90, and *p* = 0.01); and each one-point increase in the social processes score was associated with 12% higher odds of conversion (OR = 1.12, 95% CI: 1.03–1.22, and *p* < 0.01). The Hosmer–Lemeshow test was statistically significant (χ^2^ = 22.27, *p* = 0.004), suggesting suboptimal model calibration; results should be interpreted with caution. Specific data could be seen in Table 4.

### 3.6. Impact of Instant Intervention on Knowledge and Attitudes

Among the 543 participants without baseline willingness who completed the 3 min one-on-one instant educational intervention, knowledge scores increased from 3.94 ± 1.72 to 4.38 ± 1.65 (mean difference: 0.44, 95% CI: 0.31–0.57, and 11.17% increase; paired *t*-test: t = 6.60, *p* < 0.01), and attitude scores increased from 4.27 ± 0.82 to 4.35 ± 0.93 (mean difference: 0.08, 95% CI: 0.01–0.15, and 1.87% increase; paired *t*-test: t = 2.39, *p* = 0.02). Specific data could be seen in Table 5.

## 4. Discussion

Influenza vaccines in China are non-national immunization program vaccines, requiring self-payment and voluntary uptake. Annual vaccination is the most economically effective preventive measure. The elderly in Henan have a persistently low overall vaccination rate, with only 2.77% during 2020–2022 [9]. Compared with other age groups, their higher burden of chronic diseases and weaker immunity increase their susceptibility to severe influenza; moreover, the disease burden after infection is also higher than that of other age groups [12]. Given the substantial gap between high willingness (68.20%) and low uptake (6.95%) observed in this study, enhancing vaccination willingness alone is insufficient; promoting the conversion from willingness to behavior is critical for building immune barriers and protecting public health among this priority population.

In this study, the vaccination willingness among adults aged ≥60 years (68.20%) was slightly higher than that of people among adults aged ≥50 years in Henan in 2024 (68.08%), and higher than that in Chongqing (60.7%) [13] and Changsha (53.66%) [14], but lower than that of Beijing (76.61%) [15] and Shanghai (80.60%) [16]. These regional differences likely reflect disparities in economic development, health literacy, and local health promotion efforts, with more developed cities having greater public exposure to influenza- and vaccine-related information. The vaccination rate in this study was 6.95%, yielding a willingness-to-behavior conversion rate of only 9.51%—substantially lower than the 21.76% achieved in Zhejiang under its free vaccination policy. This striking disparity in uptake between Henan and Zhejiang, despite similar willingness levels (68.20% vs. 73.15%), highlights the critical role of free vaccination policies in facilitating the translation of willingness into action. Notably, the implementation of a free vaccination policy in Zhejiang significantly increased uptake, indicating that removing financial barriers is an effective strategy for closing the intention–behavior gap. However, even with a free policy, Zhejiang’s conversion rate (21.76%) [17] still left a substantial gap between willingness and behavior, suggesting that financial barriers are not the only obstacle; social and practical factors also require targeted interventions. This finding suggests that merely enhancing willingness is not sufficient for conversion into actual vaccination behavior, and more attention should be paid to the obstacles in the conversion process.

Among the BeSD dimensions, thinking and feeling was not an independent factor for either willingness or uptake in the multivariate analysis. This may reflect that, while older adults generally recognize influenza risks and the benefits of vaccination, their actual decision-making is more strongly shaped by interpersonal influences and practical constraints, which can override individual risk perceptions [10,12]. The practical issues dimension was positively associated with willingness (OR = 1.12, 95% CI: 1.06–1.19) but not with actual uptake. This discrepancy may be explained by the persistently low influenza vaccination rate in Henan (2.77–3.00%); most older adults have never been vaccinated and thus lack firsthand experience with vaccination procedures, costs, or accessibility [6]. Their understanding of practical issues may be based on hypothetical considerations rather than actual experience, leading to a gap between perceived and real barriers [7]. These findings suggest that knowledge and risk perception, while necessary, are insufficient to drive vaccination behavior without concurrent social support and practical facilitation.

The social processes dimension emerged as the strongest driver for both willingness (OR = 1.38 per 1-point increase, 95% CI: 1.33–1.44) and uptake (OR = 1.12, 95% CI: 1.03–1.22), a finding consistent with previous research in Chinese urban communities (OR = 1.15) [18]. The differential effect on willingness versus behavior—38% versus 12%—suggests that social networks operate through distinct mechanisms at different stages of the decision-making process. At the intention formation stage, family and friends primarily provide information and normative guidance, leveraging trust to shape risk perceptions and vaccination attitudes relatively quickly. However, translating intention into action requires not only attitudinal support but also tangible practical assistance, such as active encouragement from family members, reminders from village doctors or clinic staff, and community-organized vaccination services. In rural areas, where the out-migration of younger adults has left many older adults with limited immediate social support, the influence of family and friends becomes particularly pronounced. Follow-up data from this study support this mechanism, with 63.64% of initially unwilling but eventually vaccinated participants citing the influence of family/friends as the primary driver. These findings suggest that interventions should be staged: willingness formation should leverage social networks for information dissemination, while behavior conversion requires supplementary practical measures such as convenient vaccination services and reminder systems. Notably, physician recommendation also played a significant role, reported by 36.36% of these converters, highlighting the importance of professional authority within social networks.

Follow-up of the population with intention but no behavior identified three main barriers to vaccination: perceived good health (33.29%), high vaccine cost (27.12%), and lack of time (26.31%). Regarding cost and time barriers, practical solutions such as free vaccination programs, medical insurance reimbursement, and extended or weekend vaccination clinic hours during the influenza season could help reduce these obstacles. Free influenza vaccination programs have been implemented in various regions across China, with evaluations demonstrating their effectiveness in increasing vaccination coverage among older adults [19]. The predominance of “perceived good health” as the primary barrier suggests that many older adults may underestimate their susceptibility to influenza and its complications, reflecting a gap in risk perception rather than negative attitudes toward vaccination itself. This finding has important implications for health communication: interventions should move beyond simply providing factual information about influenza risks and instead adopt age-specific risk communication strategies that help older adults realistically assess their personal vulnerability, particularly in the context of underlying chronic conditions.

First, the urban–rural disparity was pronounced: urban residents had a higher willingness (OR = 1.41, 95% CI: 1.12–1.78) and higher uptake (OR = 1.64, 95% CI: 1.11–2.42) than rural residents, with the effect being stronger at the behavioral level. This pattern suggests that urban–rural gaps in both information exposure and service accessibility contribute to the disparity—urban residents benefit from greater access to health information and more convenient vaccination services, which not only shape their initial willingness but also facilitate follow-through to action. In rural areas where health information is less prevalent and vaccination services are less accessible, interventions should combine community-based health education with service delivery innovations such as mobile vaccination units and village doctor-led mobilization, integrated with routine primary health services.

Second, annual medical visit frequency was a strong predictor of uptake. Participants with ≥11 visits per year had a significantly higher uptake (OR = 2.67, 95% CI: 1.33–5.33) compared to those with ≤1 visit, while those with 2–5 visits had a lower uptake (OR = 0.59, 95% CI: 0.39–0.90). The most frequent attendees are patients with chronic conditions who require regular follow-up and thus have repeated opportunities to receive health education and vaccine recommendations. This finding supports the integration of vaccine prescriptions into routine care for chronic disease patients, shifting the paradigm from “passive treatment” to “active prevention.”

Third, current smokers had significantly lower uptake than never smokers (OR = 0.43, 95% CI: 0.22–0.85), while former smokers did not differ significantly from never smokers. This pattern suggests that smoking cessation may reflect a broader health behavior transformation that increases openness to preventive interventions.

The 3 min one-on-one instant educational intervention delivered to initially unwilling participants demonstrated modest short-term effectiveness: knowledge scores increased by 11.17%, willingness increased by 8.47% (46/543), but only 1.47% (8/543) were subsequently vaccinated. The 8.47% willingness increase was lower than the 18.5% reported by Zhang et al. (2024) [20] in a similar intervention, a difference likely attributable to the higher proportion of rural participants (50.88%) with lower health literacy, the extremely low baseline vaccination rate (2.77%), and the 28.55% loss to follow-up. The modest attitude change (1.87%) relative to the knowledge gain (11.17%) suggests that, while factual information can be transmitted relatively easily, shifting deeply held attitudes and behavioral intentions requires more sustained and multi-faceted intervention. The low conversion rate from intervention to behavior (1.47%) underscores that single-session education, while useful as an initial “touchpoint,” cannot substitute for multi-round, multi-channel, and continuous intervention strategies. Brief educational interventions should be viewed as one component of a comprehensive strategy—raising awareness and creating opportunities for follow-up—rather than a standalone solution.

The implications of this study for practical work are as follows: First, leverage social networks as the primary intervention channel. Given that family/friend influence (63.64%) and physician recommendation (36.36%) were the primary drivers among initially unwilling but eventually vaccinated participants, interventions should engage family members and community doctors as trusted vaccine advocates. Village doctors should be trained and incentivized to provide brief, targeted vaccine recommendations during routine home visits or chronic disease follow-ups. Second, tailor interventions to close the urban–rural gap. Our finding that rural residents had a significantly lower willingness (OR = 1.41) and uptake (OR = 1.64) than urban residents suggests a dual challenge of information and access. Rural strategies should integrate health education with service delivery innovations, such as mobile vaccination units timed to coincide with routine village health activities. Third, embed vaccine recommendations in clinical care for high-risk groups. The strong association between frequent outpatient visits and higher uptake (OR = 2.67 for ≥11 visits) indicates that clinical settings are critical intervention touchpoints. Clinicians should consider vaccine prescriptions as part of routine care for patients with chronic conditions, shifting the paradigm from treatment to prevention. Fourth, target smokers through coordinated interventions. Current smokers had significantly lower uptake (OR = 0.43). Integrating vaccine recommendations into smoking cessation programs could achieve a “quit-smoking + vaccination” co-intervention. Integrate brief educational interventions into routine primary care workflows as initial “touchpoints” that create awareness and willingness, but recognize that sustained, multi-channel follow-up is necessary to translate willingness into actual behavior.

This study has several limitations. First, the influenza vaccination rate among older adults in Henan is persistently low, and the sample size of the instant intervention is limited (*n* = 543), which may restrict the generalizability of the intervention-related findings. Second, the non-randomized design of the instant intervention and the exclusion of 217 initially unwilling participants (28.55%) who refused or were lost to follow-up may have introduced selection bias, potentially overestimating the intervention’s effectiveness; per-protocol results should therefore be interpreted with caution. Third, the significant Hosmer–Lemeshow test results for both the willingness and behavior models (*p* = 0.01 and *p* = 0.004, respectively) suggest that our multivariate models may not fully capture all factors influencing vaccination willingness and uptake. This lack of fit may be attributable to unmeasured confounding variables (e.g., prior influenza infection history, detailed health literacy measures), potential non-linear relationships between BeSD dimension scores and the outcomes, or interactions among BeSD dimensions not included in the main effects models. Fourth, this study combines a cross-sectional design with short-term follow-up; longer-term observational studies are needed to assess the sustainability of the intervention effects and the stability of the observed associations over time.

## 5. Conclusions

In conclusion, influenza vaccination among older adults in Henan exhibits a “high willingness, low conversion” pattern, with social processes as the strongest driver bridging the intention–behavior gap. The brief educational intervention improved knowledge and willingness but had limited impact on uptake, underscoring that isolated education is insufficient. To translate willingness into action, we recommend a multi-component strategy that leverages family/community networks, addresses urban–rural disparities, counters the “perceived good health” barrier through tailored risk communication, integrates vaccine recommendations into routine care for high-risk groups, and embeds brief interventions as initial touchpoints within sustained follow-up.

## Figures and Tables

**Table 1:** Basic characteristics of the study population and distribution of vaccination willingness and uptake.

Subject	Vaccination Willingness (*n* = 2390)				Vaccination Behavior (*n* = 1630)			
	Willing	Unwilling	χ^2^	*p*	Vaccinated	Not Vaccinated	χ^2^	*p*
age [number (%)]								
60–64	358 (70.06)	153 (29.94)	8.86	0.03	27 (7.54)	331 (92.46)	2.28	0.52
65–69	445 (71.66)	176 (28.34)			43 (9.66)	402 (90.34)		
70–74	416 (64.40)	230 (35.60)			44 (10.58)	372 (89.42)		
≥75	411 (67.16)	201 (32.84)			41 (9.98)	370 (90.02)		
educational level [number (%)]								
primary or below	522 (62.52)	313 (37.49)	49.91	<0.01	51 (9.77)	471 (90.23)	2.86	0.58
primary	441 (65.92)	228 (34.08)			42 (9.52)	399 (90.48)		
junior middle	379 (70.45)	159 (29.55)			29 (7.65)	350 (92.35)		
senior high	224 (81.75)	50 (18.25)			26 (11.61)	198 (88.39)		
college or above	64 (86.49)	10 (13.51)			7 (10.94)	57 (89.06)		
residence [number (%)]								
rural area	742 (61.02)	474 (38.98)	58.86	<0.01	54 (7.28)	688 (92.72)	7.88	<0.01
urban area	888 (75.64)	286 (24.36)			101(11.37)	787 (88.63)		
monthly income [number (%)]								
0	537 (67.38)	260 (32.62)	58.27	<0.01	41 (7.64)	496 (92.36)	4.01	0.26
0–1000 yuan	662 (62.69)	394 (37.31)			72 (10.88)	590 (89.12)		
1000–2000 yuan	130 (72.63)	49 (27.37)			11 (8.46)	119 (91.54)		
≥2000 yuan	301 (84.08)	57 (15.92)			31 (10.30)	270 (89.70)		
residence situation [number (%)]								
solitude	193 (61.08)	123 (38.92)	11.15	0.01	15 (7.77)	178 (92.23)	1.08	0.78
live only with partner	913 (69.48)	401 (30.52)			89 (9.75)	824 (90.25)		
live only with children	177 (65.31)	94 (34.69)			19 (10.73)	158 (89.27)		
live with partner and children	347 (70.96)	142 (29.04)			32 (9.22)	315 (90.78)		
chronic health problem [number (%)]								
yes	1003 (68.65)	458 (31.35)	0.35	0.55	93 (9.27)	910 (90.73)	0.17	0.68
no	627 (67.49)	302 (32.51)			62 (9.89)	565 (90.11)		
annual hospital visit frequency [number (%)]								
≤1	926 (69.26)	411 (30.74)	4.31	0.23	100 (10.80)	826 (89.20)	20.63	<0.01
2–5	557 (68.01)	262 (31.99)			33 (5.92)	524 (94.08)		
6–10	81 (64.80)	44 (35.20)			8 (9.88)	73 (90.12)		
≥11	66 (60.55)	43 (39.45)			14 (21.21)	52 (78.79)		
smoking situation [number (%)]								
never smoker	1156 (68.77)	525 (31.23)	5.46	0.07	117 (10.12)	1039 (89.88)	7.48	0.02
former smoker	254 (70.56)	106 (29.44)			28 (11.02)	226 (88.98)		
current smoker	220 (63.04)	129 (36.96)			10 (4.55)	210 (95.45)		
self-health evaluation [number (%)]								
well	678 (70.33)	286 (29.67)	4.00	0.14	61 (9.00)	617 (91.00)	0.36	0.84
fair	793 (67.20)	387 (32.80)			78 (9.84)	715 (90.16)		
poor	159 (64.63)	87 (35.37)			16 (10.06)	143 (89.94)		

**Table 2:** BeSD dimension scores by vaccination willingness and uptake.

BeSD Dimension	Vaccination Willingness (*n* = 2390)				Vaccination Behavior (*n* = 1630)			
	Willing	Unwilling	*t*	*p*	Vaccinated	Not Vaccinated	*t*	*p*
thinking and feeling								
number	1630	760	13.84	<0.01	155	1475	4.37	<0.01
score	22.13 ± 4.32	19.49 ± 4.41			23.57 ± 3.90	21.98 ± 4.34		
social processes								
number	1630	760	29.21	<0.01	155	1475	7.51	<0.01
score	15.98 ± 3.22	11.45 ± 3.67			17.46 ± 2.51	15.82 ± 3.25		
practical issues								
number	1630	760	20.86	<0.01	155	1475	6.60	<0.01
score	12.41 ± 2.52	10.12 ± 2.46			13.49 ± 2.11	12.29 ± 2.53		

**Table 3:** Multivariate logistic regression analysis of the vaccination willingness survey.

Variable	B	SE	Wald χ^2^	OR (95%CI)	*p*
residence (compared with rural area)					
urban area	0.35	0.12	8.74	1.41 (1.12–1.78)	<0.01
monthly income (compared with 0 yuan)					
0–1000 yuan	−0.24	0.12	3.87	0.79 (0.62–1.00)	0.049 <0.05
1000–2000 yuan	−0.26	0.22	1.36	0.77 (0.50–1.20)	0.24
≥2000 yuan	0.35	0.22	2.61	1.42 (0.93–2.17)	0.11
score of thinking and feeling dimension	−0.02	0.02	1.88	0.98 (0.95–1.01)	0.17
score of social processes dimension	0.32	0.02	270.22	1.38 (1.33–1.44)	<0.01
score of practical issues dimension	0.12	0.03	16.25	1.12 (1.06–1.19)	<0.01

**Table 4:** Multivariate logistic regression analysis of the vaccination behavior survey.

Variable	B	SE	Wald χ^2^	OR (95%CI)	*p*
residence (compared with rural area)					
urban area	0.49	0.20	6.09	1.64 (1.11–2.42)	0.01
annual hospital visit frequency (compared with ≤1)					
2–5	−0.53	0.22	6.10	0.59 (0.39–0.90)	0.01
6–10	0.20	0.40	0.25	1.22 (0.55–2.70)	0.62
≥11	0.98	0.35	7.69	2.67 (1.33–5.33)	<0.01
smoking situation (compared with never smoker)					
former smoker	0.08	0.23	0.12	1.08 (0.690–1.70)	0.73
smoker	−0.83	0.34	5.91	0.43 (0.22–0.85)	0.02
score of thinking and feeling dimension	0.02	0.03	0.65	1.02 (0.97–1.08)	0.42
score of social processes dimension	0.12	0.04	7.08	1.12 (1.03–1.22)	<0.01
score of practical issues dimension	0.06	0.05	1.28	1.06 (0.96–1.18)	0.26

**Table 5:** Evaluation of intervention effectiveness (*n* = 543).

Subject		Total Score	*t*	*p*
knowledge	before intervention	3.94 ± 1.72	6.60	<0.01
	after intervention	4.38 ± 1.65		
attitude	before intervention	4.27 ± 0.82	2.39	0.02
	After intervention	4.35 ± 0.93		

## Data Availability

Data available on request due to privacy restrictions.

## Supplementary Materials

The following supporting information can be downloaded at: https://www.mdpi.com/article/10.3390/vaccines14070605/s1, Table S1. Regression Analysis Variable Assignment of the Vaccination Willingness Survey. Table S2. Regression Analysis Variable Assignment of the Vaccination Behavior Survey.

## Abbreviations

The following abbreviations are used in this manuscript:

WHO	World Health Organization
BeSD	Behavioral and Social Drivers
CDC	Center for Disease Control and Prevention
