# Supplementary material for: Influenza Vaccination Willingness, Uptake, and Behavioral Drivers Among Adults Aged ≥60 Years in Henan Province: A BeSD-Based Survey with Registry Follow-Up
Source: Vaccines (Basel). 2026 Jul 9;14(7):605. doi: 10.3390/vaccines14070605 (PMC13419047; doi:10.3390/vaccines14070605)
Supplement: Supplementary file 1 [file vaccines-14-00605-s001.zip › vaccines-4367312-supplementary.pdf]

Table S1. Regression Analysis Variable Assignment of the Vaccination Willingness Survey

variable	assignment
age	60-64 =1，65-69=2，70-74=3，≥75=4
educational level	primary or below =1，primary =2，junior middle =3，senior high =4，college or above =5
residence	rural area =1，urban area =2
monthly income	0yuan=1，0-1000yuan=2，1000-2000yuan=3，≥2000yuan=4
residence situation	live alone =1，live only with spouse =2，live only with children=3，live with spouse and children =4

Table S2. Regression Analysis Variable Assignment of the Vaccination Behavior Survey

variable	assignment
**residence**	rural area =1，urban area =2
**annual hospital visit frequency**	≤1=1，2-5=2，6-10=3，≥11=4
**smoking situation**	never smoker =1，former somker =2，somker =3
